# How to find simple and accurate rules for viral protease cleavage specificities

**DOI:** 10.1186/1471-2105-10-149

**Published:** 2009-05-16

**Authors:** Thorsteinn Rögnvaldsson, Terence A Etchells, Liwen You, Daniel Garwicz, Ian Jarman, Paulo JG Lisboa

**Affiliations:** 1 Embedded and Intelligent Systems, Halmstad University, SE-30118, Halmstad, Sweden; 2 AASS, Örebro University, SE-70182, Örebro, Sweden; 3 School of Computing and Mathematical Sciences, Liverpool John Moores University, Liverpool, L3 5UH, UK; 4 Department of Theoretical Physics, Lund University, SE-22362, Lund, Sweden; 5 Division of Clinical Chemistry and Blood Coagulation, Department of Molecular Medicine and Surgery, Karolinska Institutet, Karolinska University Hospital, SE-17176, Stockholm, Sweden; 6 Division of Clinical Chemistry and Pharmacology, Department of Medical Sciences, Uppsala University, Akademiska Sjukhuset (Uppsala University Hospital), SE-751 85, Uppsala, Sweden

## Abstract

**Background:**

Proteases of human pathogens are becoming increasingly important drug targets, hence it is necessary to understand their substrate specificity and to interpret this knowledge in practically useful ways. New methods are being developed that produce large amounts of cleavage information for individual proteases and some have been applied to extract cleavage rules from data. However, the hitherto proposed methods for extracting rules have been neither easy to understand nor very accurate. To be practically useful, cleavage rules should be accurate, compact, and expressed in an easily understandable way.

**Results:**

A new method is presented for producing cleavage rules for viral proteases with seemingly complex cleavage profiles. The method is based on orthogonal search-based rule extraction (OSRE) combined with spectral clustering. It is demonstrated on substrate data sets for human immunodeficiency virus type 1 (HIV-1) protease and hepatitis C (HCV) NS3/4A protease, showing excellent prediction performance for both HIV-1 cleavage and HCV NS3/4A cleavage, agreeing with observed HCV genotype differences. New cleavage rules (consensus sequences) are suggested for HIV-1 and HCV NS3/4A cleavages. The practical usability of the method is also demonstrated by using it to predict the location of an internal cleavage site in the HCV NS3 protease and to correct the location of a previously reported internal cleavage site in the HCV NS3 protease. The method is fast to converge and yields accurate rules, on par with previous results for HIV-1 protease and better than previous state-of-the-art for HCV NS3/4A protease. Moreover, the rules are fewer and simpler than previously obtained with rule extraction methods.

**Conclusion:**

A rule extraction methodology by searching for multivariate low-order predicates yields results that significantly outperform existing rule bases on out-of-sample data, but are more transparent to expert users. The approach yields rules that are easy to use and useful for interpreting experimental data.

## Background

The human body contains almost 600 proteases [1] that are involved in a number of important biological pathways such as blood coagulation, fibrinolysis, complement activation, hormone production and digestion [2]. These proteases are often essential players in elaborate networks, or cascades, where zymogens (catalytically inactive protease precursors) are activated in order to activate a downstream zymogen or digest/inactivate a structural or biologically active protein. Proteases therefore tend to have highly specific substrate repertoires and are regulated by endogenous protease inhibitors, with a delicate balance between these counteracting forces. An imbalance between active proteases and protease inhibitors may cause, or contribute to, many diseases. A classical example is hereditary deficiency of *α*1 proteinase inhibitor (also known as antitrypsin), that can lead to early-onset emphysema [3]. This process is greatly accelerated upon smoking, partly because antitrypsin is susceptible to inactivation by oxidation by cigarette smoke and because neutrophil elastase, a potent protease with a broad substrate specificity including lung elastin, is up-regulated and released from neutrophils by nicotine. Similarly, the progression of some severe diseases can be successfully slowed down with the use of protease inhibitors. Proteases are consequently important drug targets [4] and the list of protease inhibitors approved for clinical use is steadily growing, including drugs to treat, e.g., hypertension, thrombosis, pancreatitis, periodontitis, respiratory diseases, cancer, HIV/AIDS and probably soon hepatitis C [5]. However, the road to success has been paved with numerous failures, because of many unexpected (and in most cases unwanted) side effects. An ideal protease inhibitor should have a well defined substrate specificity, broad enough to treat the disease efficiently, but narrow enough not to interfere with other proteases or biological processes, combined with low toxicity to the host cells. HIV-1 protease has an important function for virus maturation during HIV infection, but its substrate specificity is complex and endogenous protease inhibitors of the host cells infected with HIV do not seem to be able to stop its action. Therefore, artificial HIV-1 protease inhibitors are needed and have been in clinical use for HIV/AIDS treatment for over a decade. Among other viral targets, the hepatitis C virus (HCV) NS3 protease is also a candidate target for antiviral drugs, since currently available HCV therapy is effective in only half of the patients and limited by serious side effects [6,7].

A challenge in the hunt for better protease inhibitors is to make maximum use of available experimental protease data and provide accurate rules for the substrate specificity, rules that can be used to estimate the effect of proteases in biological pathways. There are three important criteria that should be met by these specificity rules: they should be accurate in terms of out-of-sample prediction; they should be comprehensible (understandable); and they should have a high fidelity to the data from which they were extracted. The aim of the present work was to present a method for extracting cleavage rules from viral protease data. A method that provides rules that meet these three criteria better than previously presented approaches. The method is exemplified on HIV-1 and HCV NS3 protease data sets.

### The structure of cleavage rules

The first step towards meeting the three requirements above (accuracy, comprehensibility, and fidelity) is to understand how cleavage rules are typically formulated. Rules that are expressed in the standard form are probably more likely to be comprehensible, and they may even be more accurate if the standard form has developed over time so that people have included their knowledge of how protease cleavage works into these rules.

Cleavage specificities tend to be described assuming that certain positions should contain certain amino acids. Common terms used are "consensus motif" (or "consensus sequence") and "sub-site preference" (see e.g. [8]), where the latter refers to those amino acids that seem to match the active site in the protease, and sub-site preferences are typically illustrated with specificity profiles or histograms showing the frequency of amino acids in the different positions of cleaved substrates. This assumption is evident in the rules used for different proteases in the tools PeptideCutter and PeptideMass on ; they are all expressed as lists of allowed (or disallowed) amino acids in the positions of the peptides (examples are provided below).

The tradition of expressing cleavage rules in this fashion may not be a coincidence, and a rule extraction method that explicitly produces conjunctive rules may therefore be the right way to go for these problems.

Conjunctive rules are rules that are formulated as lists of requirements that must all be true (i.e. the requirements for each position are connected with a logical AND). Using conjunctive rules puts no restriction on the possible cleavage specificities that can be described; conjunctive rules can be combined with the logical OR function into any logical rule. This is the so-called Disjunctive Normal Form Theorem [9].

A very simple cleavage rule is, e.g., the Arg-C proteinase cleavage rule [10]. It cleaves peptides on the C-terminal side of Arginine (Arg, R). This is expressed as



using the standard Schechter and Berger notation [11] where the substrate sites are denoted by ...-P3-P2-P1-P1'-P2'-P3'-..., with the scissile bond located between P1 and P1'.

An effective immune response of the human host against a viral attack involves the generation of virus-specific cytotoxic T lymphocytes (CTLs), whose main function is to kill virus-infected cells. The two important countermeasures used by the CTLs are perforin/granzyme mediated apoptosis and Fas-mediated apoptosis. Granzyme B, a member of the hematopoietic serine protease superfamily, is stored in granules of cytotoxic T cells and natural killer cells and has a requirement for Aspartate (Asp, D) in the P1 position [12]. However, the full rule is a conjunctive rule that involves more positions [13]:



which means that it cleaves on the C-terminal side of the sequence IEPD, Isoleucine-Glutamate-Proline-Aspartate (Ile-Glu-Pro-Asp), i.e. all four positions must have specific amino acids in them for cleavage to occur.

Each position can also allow a set of amino acids, as is the case for the bovine coagulation factor, Factor Xa [14]:



This means that position P4 should be occupied by any of {A,G,I,L,T,V,M}, but nothing else, that position P3 should be occupied by any of {D,E}, but nothing else, that P2 should be occupied by G, and that P1 should be occupied by R for cleavage to occur (and it occurs on the C-terminal side of R).

There are also proteases that have more than one rule, e.g. if there are two types of cleavages that occur. This is the case for Thrombin [10], which has the following two cleavage rules:



and



The notation for positions P1' and P2' means that cleavage will not occur if Aspartate (Asp, D) or Glutamate (Glu, E) are in any of those positions.

Combinations of conjunctive rules can also be used to describe cleavage rules if there are interactions and competitions among the positions. Assume for instance a cleavage site with two nearby positions (P2 and P1) with preference for hydrophobic amino acids, e.g. L, M, F, Y (Leucine, Methionine, Phenylalanine and Tyrosine). Assume also that it is impossible to fit both positions with F or Y at the same time (due to space constraints) but that it is sufficient that one of them is present in one of the two positions. This would lead to a set of two conjunctive rules



and



To conclude, conjunctive rules follow the notation used in standard tools for describing cleavage of peptides and they can be used to describe any type of rule. If conjunctive rules can be used to produce cleavage rules that are simpler but as accurate as those from other methods that do not follow the standard notation, then that would speak in favour of a conjunctive rule approach.

## Results

### Data

#### HIV-1 protease data

You et al. [15] collected a HIV-1 protease substrate data set containing 746 octamers, of which 401 are cleaved and 345 are non-cleaved. In this data set, the octamer SQNYAIVQ was originally erroneously labeled as non-cleaved [16]. This error was corrected and the resulting data set is referred to as the HIV-1 PR 746 data set in this study. The octamers are denoted P4-P3-P2-P1-P1'-P2'-P3'-P4'.

Kontijevskis et al. [17] collected a substantially larger HIV-1 protease substrate data set containing 1625 octamers, where 374 are cleaved and 1251 are non-cleaved. This data set also has the wrong label for SQNYAIVQ, which we corrected. The corrected data set is referred to as the HIV-1 PR 1625 data set in this study.

The similarity between the HIV-1 PR 746 and the HIV-1 PR 1625 data sets is very high since they were partly collected from the same literature sources. There are 659 octamers that are common between the HIV-1 PR 746 and the HIV-1 PR 1625 data sets. Of these, seven are labeled differently in the two data sets: AAAMSSAI, ARVLAQAM, GRINVALV, SGVFSVNG and SGVYQLSA are labeled as cleaved in the 746 data set and as non-cleaved in the 1625 data set; AEAMSQVT and FRSGVETT are labeled as non-cleaved in the 746 data set and as cleaved in the 1625 data set.

Recently, Schilling and Overall [8] collected an even larger set of peptides cleaved by HIV-1 protease. This set was published after the rules described herein were constructed and can thus be used as independent test data. A set of octamers was generated from this data in the following way: cleaved octamers were taken from all peptides that contained at least four residues on the prime and the non-prime sides of the scissile bond; non-cleaved octamers were generated by sliding a window of size eight residues on both sides of the scissile bond (but not across the scissile bond) if the prime or the non-prime side had at least eight residues. For example, Schilling and Overall [8] (supplementary Table nineteen) report the cleaved peptide PLLGGSLMEYAILSAIAAMNEPK, where the cleavage site is between Y and A. This produces the cleaved octamer LMEYAILS and the non-cleaved octamers PLLGGSLM, LLGGSLME, LGGSLMEY, AILSAIAA, ILSAIAAM, LSAIAAMN, SAIAAMNE, AIAAMNEP and IAAMNEPK. Peptides that had an ambiguous P1 residue or that were marked "excluded" in Tables nineteen and twenty in the supporting material to their paper [8] were removed.

The final data set contains 3261 octamers, of which 436 are cleaved and 2825 are non-cleaved. We refer to this as the HIV-1 PR 3261 data set.

There is no overlap between the HIV-1 PR 746 and the HIV-1 PR 3261 data sets. There are twenty common octamers in the HIV-1 PR 1625 and the HIV-1 PR 3261 data sets, of which two are in conflict (EENFAVEA and QEEMLQRE, which are both labeled as non-cleaved in the 1625 data set but as cleaved in the 3261 data set). There is only one sequence in the HIV-1 PR 3261 data set that differs by one position from any octamer from any sequence in the HIV-1 PR 746 or HIV-1 PR 1625 data sets. This sequence is GWVLGEHG, which is labeled as cleaved and differs in one position from the cleaved GWILGEHG in the HIV-1 PR 746 and HIV-1 PR 1625 data sets. There are two sequences in the HIV-1 PR 746 data set that differ in two positions from sequences in the HIV-1 PR 3261 data set: the cleaved ARVLFDAL, which is similar to the non-cleaved APVLLDAL in the HIV-1 PR 3261 data set, and the cleaved GWILAEHG, which is similar to the cleaved GWVLGEHG in the HIV-1 PR 3261 data set. There are four sequences in the HIV-1 PR 1625 data set that differ in two positions from sequences in the HIV-1 PR 3261 data set: the non-cleaved NKILLAEL, VDKLVSAG and TEEKIKAL, which are similar to the non-cleaved octamers NKVNLAEL, VDVLVSSG and TEDKINAL, and the cleaved octamer GWILAEHG that differs in two positions from the cleaved GWVLGEHG in the HIV-1 PR 3261 data set. All other sequences in the HIV-1 PR 3261 data set differ in at least three positions out of eight from any octamer in the HIV-1 PR 746 or the HIV-1 PR 1625 data sets.

It is also relevant to check how many sequences in the data sets that have identical residues in the most important positions in the octamer: P2-P1-P1'-P2'. About 92% of the octamers in the HIV-1 PR 746 data set are identical, in the four central positions, to sequences in the HIV-1 PR 1625 data set. However, only 7% of the octamers in the HIV-1 PR 746 data set match to sequences in the HIV-1 3261 data set. About 41% of the octamers in the HIV-1 PR 1625 data set are identical to sequences in the HIV-1 PR 746 data set, but only 7% of the octamers in the HIV-1 PR 1625 data set match sequences in the HIV-1 PR 3261 data set. About 1% of the sequences in the HIV-1 PR 3261 data set match to sequences in the HIV-1 PR 746 data set, and 4% match to sequences in the HIV-1 PR 1625 data set.

There is thus very little sequence similarity between the HIV-1 PR 3261 data set and the HIV-1 PR 746 or the HIV-1 PR 1625 data sets, while there is a lot of sequence similarity between the two smaller data sets. The smaller data sets are used to extract cleavage rules, which are then tested on the larger (3261) data set.

#### HCV protease data

We initially intended to use a HCV NS3 protease data set used previously by other researchers [18-22]. There were, however, several uncertainties and conflicts between this data set and available references on HCV NS3 cleavage [23-30], which made us doubt the quality of this data set. A new HCV NS3 data set was therefore created from scratch from the references. A procedure described by [18] was followed to generate 706 additional non-cleaved decamers by moving a ten residue window over the 4B, 5A and 5B non-structural proteins [31] of the HCV polyprotein [GenBank: AJ238799], excluding the cleavage sites themselves since they were already in the data set. The 4A part was excluded since it is a protease co-factor. The decamers are of the form P6-P5-P4-P3-P2-P1-P1'-P2'-P3'-P4', i.e. the cleavage occurs between position six and seven in the decamer. The final HCV NS3 data set contains 939 decamers, of which 199 are cleaved and 740 are non-cleaved. We denote this data set the HCV NS3 data set.

The HCV NS3 data set is quite different from the data set used in previous rule extraction studies [18-22]: 8% of the decamers that occur in both sets are labeled differently.

Three separate out-of-sample test data sets for HCV were created. The NS3 protease itself [GenBank: NP_803144] was used to generate one test data set with 621 decamers, of which none are in the HCV NS3 data set. This test data set was intended for comparison with reported internal cleavage sites in the NS3 protease [32-34]. Four proteins from the TLR3 pathway were used for another test data set: I*κ*B kinase *ε *(IKK*ε*) [GenBank: AAC51216]; TRAF family member-associated NF-*κ*B activator-binding kinase 1 (TBK1) [GenBank: NP_037386]; Toll-like receptor 3 (TLR3) [GenBank: NP_003256]; and Toll-IL-1 receptor domain-containing adaptor inducing IFN-*β *(TRIF or TICAM-1) [GenBank: BAC55579]. These four proteins have been tested for HCV NS3 cleavage by Li et al. [35]. The four proteins produced a total of 2805 decamers, of which one is also in the HCV NS3 data set (the only observed cleavage site in TRIF). A third test data set was made up of 69 *in vivo *tested NS3 substrates from Kim et al. [36], none of which are in the HCV NS3 data set. The three out-of-sample test sets are denoted NS3 internal, TLR3, and NS3 in vivo, respectively.

#### Artificial data

Two artificial data sets were created to measure the orthogonal search-based rule extraction (OSRE) method's ability to extract rules of the form that we are looking for. The data sets were designed using conjunctive rules to mimic typical cleavage rules as described in the introduction.

The two artificial rule sets are shown in Table 1. The simpler problem (A) was modeled partly after the Thermolysin [10] specificity, as described for the PeptideCutter tool. Four positions were used instead of the minimum two in order to see how well OSRE could deal with irrelevant information. The more complex problem (B) was modeled after the Thrombin [10] specificity, as described for the PeptideCutter tool. Training peptides from data set A were sampled randomly. Training peptides from data set B were picked with balanced sampling (i.e. achieving a 1:1 ratio of cleaved to non-cleaved sequences). This was done because the random probability for observing a cleaved peptide for data set B is very low.

**Table 1:** Artificial data set.

Artificial data set A (4-mers: P2-P1-P1'-P2')	Artificial data set B (6-mers: P4-P3-P2-P1-P1'-P2')
P1' ∈ {A,I,L,M,F,V}	P4 ∈ {A,G,I,L,M,F,T,V}
P2' ∉ {P}	P3 ∈ {A,G,I,L,F,T,V,W}
	P2 ∈ {P}
	P1 ∈ {R}
	P1' ∉ {D,E}
	P2' ∉ {D,E}

Rules for the two artificial data sets used. occur.

Any amino acid could occupy the two first positions in artificial data set A (the generated peptides were longer than the actual rule). One letter amino acid abbreviations are used. The sign ∈ means "in" and the sign ∉ means "not in". The rules are connected with the Boolean AND operator, which means that all position rules must be true for cleavage to

### The power of OSRE – the artificial data

Table 2 lists the OSRE rules extracted for the two artificial protease specificity problems for different sizes of the training data sets. Problem A is easy but problem B is quite a lot trickier. OSRE quickly finds the rule (P2 ∈ {P} AND P1 ∈ {R}) and this simple rule is sufficient to get very high classification accuracy (approximately 99.7% correct) on the data. A huge amount of additional data is then required before the full rule is extracted. This is because a very low fraction of the negative examples are in conflict with this rule. OSRE extracts the almost correct rule when it is presented with a data set with 100,000 examples.

**Table 2:** OSRE performance on artificial data set.

Artificial data set A		Artificial data set B	
Data	OSRE rule	Data	OSRE rule

10	P2 ∈ {D,E,G,S,W,V}	10^2^	P2 ∈ {P}
			P1 ∈ {R}

10^2^	P1' ∈ {A,I,L,M,F,V}	10^3^	P4 ∉ {S}
			P2 ∈ {P}
			P1 ∈ {R}

10^3^	P1' ∈ {A,I,L,M,F,V}	10^4^	P4 ∉ {R,N,Q,E,K,S}
	P2' ∉ {P}		P3 ∈ {Q}
			P2 ∈ {P}
			P1 ∈ {R}

		10^5^	P4 ∈ {A,G,I,L,M,F,T,V}
			P3 ∈ {A,G,I,L,F,T,V,W}
			P2 ∈ {P}
			P1 ∈ {R}
			P1' ∉ {D,E}

Rules extracted with OSRE from the artificial data sets (cf. Table 1). The left column for each data set shows the number of peptides used to extract the rules.

### HIV-1 protease

#### Rule extraction

**Figure 1:**
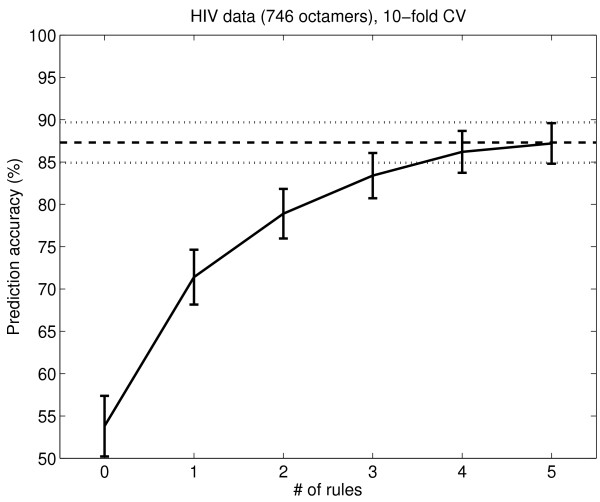
**OSRE rules' performance on the HIV-1 PR 746 data set**. The performance of the OSRE rules on the HIV-1 PR 746 data. The x-axis shows the number of rules used in the prediction. The y-axis shows the CV accuracy. The error bars are 1.96 times the binomial standard deviations. The horizontal lines show the accuracy when all rules (even more than 5) are used. The accuracy for zero rules is the default accuracy, when all peptides are classified as the majority class.

**Figure 2:**
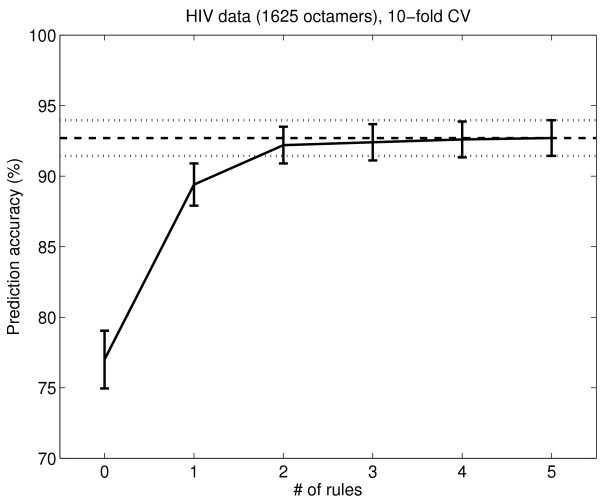
**OSRE rules' performance on the HIV-1 PR 1625 data set**. The performance of the OSRE rules on the 1625 peptide HIV-1 PR data. The x-axis shows the number of rules used in the prediction. The y-axis shows the CV accuracy (%). The error bars are 1.96 times the binomial standard deviations. The horizontal lines show the accuracy when all rules (even more than 5) are used. The accuracy for zero rules is the default accuracy, when all peptides are classified as the majority class.

Rules for the HIV-1 PR 746 and HIV-1 PR 1621 data sets were extracted using OSRE, as described in the Methods section. OSRE produced slightly different numbers of rules for each cross validation (CV) subset, varying between 7 and 10 rules for the 746 peptide data set and between 6 and 9 rules for the 1625 peptide data set. The CV generalization error was estimated for the rules when one, two, three, four, five and all rules were used to predict cleavage for the hold-out CV data set (rules were ordered in priority order by OSRE); this CV error is shown in Figure 1 and Figure 2. The 746 peptide set is a bit more difficult to predict because it is a balanced data set with fewer negative examples than the 1625 peptide set. The CV performance improves until five rules are used (this was one motivation for using five clusters in the rule clustering). The CV prediction accuracies of the OSRE method when using all rules are 87% for the 746 peptide data set and 93% for the 1625 peptide set.

The fidelity is measured by using the rules to label the peptide data sets from which they were generated. The OSRE consensus rules, produced by using spectral clustering on the OSRE rule sets (as described in the Methods section), are listed in Table 3. These consensus rules were used to label the HIV-1 PR peptides in the data sets and the resulting in-sample accuracy (we denote this the fidelity) is shown in Table 4. Table 4 also lists, for reference, the fidelities for rule sets generated using the rough set theory approach [17].

**Table 3:** Consensus rules for the HIV-1 PR data sets.

HIV-1 PR 746 peptide set		HIV-1 PR 1625 peptide set	
HIVA1	P3 ∉ {N}	HIVA2	P4 ∉ {N,C,I}
	P2 ∉ {Q,L,K,S}		P2 ∉ {Q,K,P,S}
	P1 ∈ {G,L,M,F,Y}		P1 ∈ {L,M,F,Y}
	P1' ∈ {H,I,L,M,F,P,Y,V}		P1' ∉ {R,N,D,C,Q,E,K,S,T}
	P2' ∉ {R,N,D,G,H,K,P,S}		P2' ∉ {R,N,D,G,H,K}
			P3' ∉ { K }

HIVB1	P1 ∉ {Q,K,T,V}	HIVB2	P4 ∉ {N,C,I,K,M,F,W,Y}
	P1' ∈ {L,F,P,Y}		P3 ∉ {N,C,V}
	P2' ∉ {R,N,D,G,H,K,S,T,Y}		P2 ∉ {K}
	P3' ∉ {N,H,I,L,K,P,S}		P1 ∉ {R,Q,E,K,T}
	P4' ∉ {N,Q,I,M,V}		P1' ∉ {R,N,D,C,Q,E,K,S,T}
			P2' ∈ {A,C,Q,E,I,L,S,V}
			P3' ∈ {A,C,Q,M,F,Y}

HIVC1	P2 ∉ {Q,L,K,S}	HIVC2	P4 ∉ {N,C,I,K,M,F,W,Y}
	P1 ∉ {K,T,V}		P3 ∉ {N,V}
	P2' ∈ {E}		P2 ∈ {A,C,I,M,F,V}
	P3' ∉ {N}		P1 ∉ {R,Q,E,K,P,T}
			P1' ∉ {R,N,D,C,Q,E,K,S,T}
			P2' ∈ {A,C,Q,I,L,S,V}
			P3' ∉ {N,K,P}

HIVD1	P3 ∉ {N,C,S,V}	HIVD2	P4 ∉ {C,I,Y}
	P2 ∈ {I,V}		P3 ∉ {N,V}
	P1 ∉ {Q,K,P,T,V}		P2 ∈ {A,C,I,M,F,T,V}
	P1' ∉ {D,Q,K,S}		P1 ∈ {A,L,M,F,Y}
	P2' ∉ {R,N,D,G,K}		P1' ∉ {K}
	P3' ∉ {N}		P2' ∈ {A,E,I,L,S,V}
	P4' ∉ {A,N,Q,I,M,W,Y,V}		P3' ∉ {N,I,K,P}

HIVE1	P1 ∈ {L,M,F,Y}	HIVE2	P2 ∈ {A,C,I,M,F,V}
	P1' ∈ {L,M,F,P,Y}		P1 ∉ {R,Q,E,K}
	P4' ∉ {A,N,I,M,Y,V}		P2' ∈ {A,C,Q,E,I,L,S,T,V}
			P3' ∈ {A,C,M,F,Y}

Consensus rules for the HIV-1 PR data sets. The rules are listed in the priority order given by the OSRE method. The text in italics is the label for each rule.

**Table 4:** Fidelity for HIV-1 PR rules.

Rules used	Accuracy 746 HIV-1	Accuracy 1621 HIV-1
HIVA1, HIVB1, ..., HIVE1	92.9%	90.0%
HIVA2, HIVB2, ..., HIVE2	92.2%	94.9%
[17] Fig. 1a	63.4%	85.4%
[17] Fig. 1b	75.3%	65.2%
[17] Table III	63.0%	85.1%
[17] Table IV	75.5%	65.3%

The fidelity of the rules for the HIV-1 PR data sets (cf. Table 3).

#### Out-of-sample tests

The OSRE rules' prediction accuracy was tested on the HIV-1 PR 3261 data set. This data set was published after the rules were extracted and there is almost no sequence overlap with the data set used to generate the rules. It therefore constitutes a true out-of-sample test of the rules' ability to predict cleavage for novel sequences. However, the HIV-1 PR 3261 data set has many more non-cleaved octamers than cleaved octamers; the prediction accuracy is almost 87% if all sequences are predicted as non-cleaved (which is not a very useful prediction). The prediction accuracy is therefore not a good quality measure for the performance of the rules, it is better to present the sensitivity (true positive fraction), specificity (true negative fraction), and positive likelihood ratio. These are defined as:

(1)

(2)

(3)

The positive likelihood ratio measures how much better the odds of correctly predicting a cleavage location with the rule set are than predicting randomly according to prevalence.

**Figure 3:**
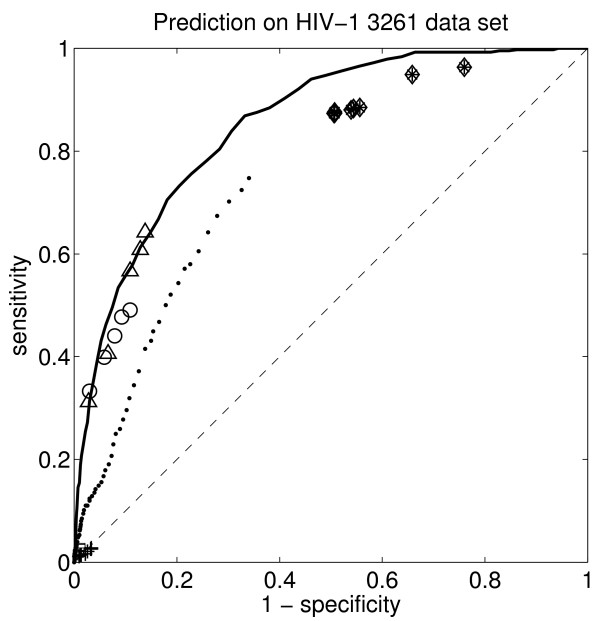
**The receiver operator characteristic (ROC) for the OSRE rules’ accuracy on the out-of-sample HIV-1 PR 3261 data set**. The triangles show the results for the OSRE HIV-1 PR 746 rules and the circles show the results for the OSRE HIV-1 PR 1625 rules (the five symbols correspond to results using 1, 2, 3, 4, and 5 rules, respectively). The diamonds and stars show the rough set rules’ prediction accuracy, Table III and Fig. 1b in [17]. The diamonds and stars show the results when 1, 2, 3, ..., and 9 rules are used from [17]. The squares and the crosses in the detail Figure, Fig. 4, are the results for Table IV and Fig. 1a in [17]. The dots show the prediction accuracy for the HIVcleave web-server [37]. The solid curve shows the ROC curve for the hitherto best predictor, a linear support vector machine trained on the HIV-1 PR 1625 data set [38], which does not provide any rules. The dashed diagonal line marks the expected results for random prediction.

**Figure 4:**
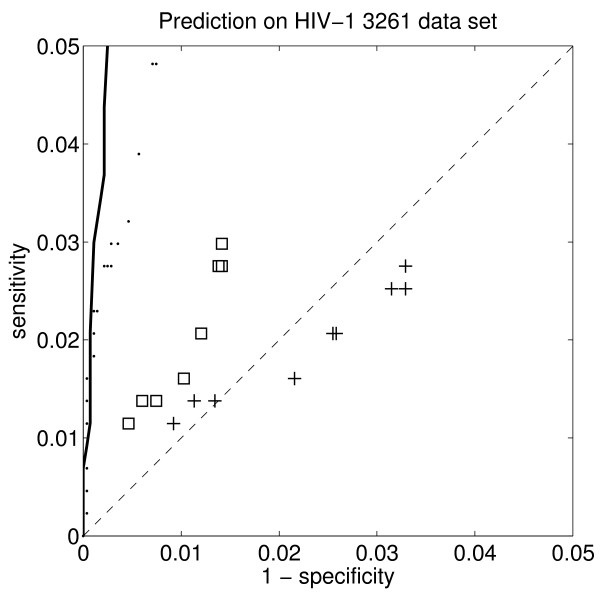
**ROC for different rule based systems on the out-of-sample HIV-1 PR 3261 – detail**. Detail of the receiver operator characteristic (ROC) for the OSRE rules' accuracy on the out-of-sample HIV-1 PR 3261 data set. The squares and the crosses are the results for Table 4 and Fig. 1a in [17]. The squares show the results when using 1, 2, 3, ..., and 10 rules from Fig. 1a in [17] and the crosses for Table 4 in [17]. The dots show the prediction accuracy for the HIVcleave web-server [37]. The solid curve shows the ROC curve for the hitherto best predictor, a linear support vector machine trained on the HIV-1 PR 1625 data set [38], which does not provide any rules. The dashed diagonal line marks the expected results for random prediction.

The sensitivity and specificity are usually shown together in a so-called receiver operator characteristic (ROC) curve. The ROC plots for the OSRE HIV-1 rules are shown in Figure 3 and Figure 4, together with the same values for the rough set theory rules [17], the recently published HIVcleave web-server [37], and a linear support vector machine [38], which was the best predictor we had hitherto tried for this problem. The positive likelihood ratio is shown in Table 5, together with corresponding sensitivity and specificity values.

**Table 5:** Positive likelihood ratios for HIV-1 PR rules.

Rules used	Positive likelihood ratio	Sensitivity	Specificity
HIVA1	11.3	31%	97%
HIVA1, HIVB1	6.2	41%	93%
HIVA1, HIVB1, HIVC1	5.2	57%	89%
HIVA1, HIVB1, HIVC1, HIVD1	4.7	61%	87%
HIVA1, HIVB1, HIVC1, HIVD1, HIVE1	4.7	64%	86%

HIVA2	11.3	33%	97%
HIVA2, HIVB2	6.8	40%	94%
HIVA2, HIVB2, HIVC2	5.6	44%	92%
HIVA2, HIVB2, HIVC2, HIVD2	5.2	48%	91%
HIVA2, HIVB2, HIVC2, HIVD2, HIVE2	4.5	49%	89%

Kontijevskis et al. [17] Fig. 1a	2.1	3%	99%
Kontijevskis et al. [17] Fig. 1b	1.7	87%	49%
Kontijevskis et al. [17] Table III	0.8	3%	97%
Kontijevskis et al. [17] Table IV	1.7	87%	49%

HIVcleave [37]	45.4	2%	100%
HIVcleave [37]	3.0	32%	89%
HIVcleave [37]	2.8	50%	82%

L-SVM [38]	29.1	2%	100%
L-SVM [38]	10.6	31%	97%
L-SVM [38]	6.7	50%	93%

The positive likelihood ratios for different rules and predictors on the HIV-1 3261 data set. The results are shown for the OSRE HIV-1 rules and the Kontijevskis et al. rough set theory rules [17], together with some reference values for the HIVcleave web server [37] and a linear support vector machine [38].

### HCV NS3 protease

#### Rule extraction

**Figure 5:**
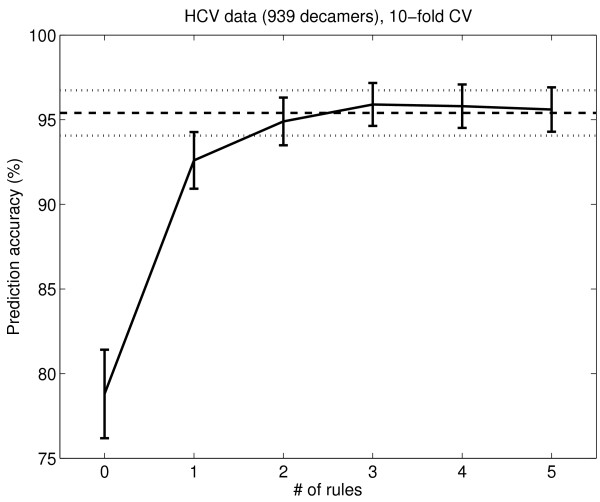
**Performance of OSRE rules on the HCV NS3 data set**. The performance of the OSRE rules on the 939 peptide HCV NS3 data. The x-axis shows the number of rules used in the prediction. The y-axis shows the out-of-sample accuracy (%). The error bars are 1.96 times the binomial standard deviations. The horizontal lines show the accuracy when all rules are used. The accuracy for zero rules is the default accuracy, when all peptides are classified as the majority class.

The OSRE method was applied to the HCV NS3 data in a corresponding way as for the HIV-1 PR data. The CV out-of-sample error as a function of the number of rules is shown in Figure 5. The accuracy when using all rules is 95%. This is close to the performance of a non-rule-based linear support vector machine classifier with tuned "slack", which has an accuracy of 97%, and it is the hitherto best result using a rule based method for HCV NS3. The consensus rules for HCV NS3 protease are listed in Table 6. Their fidelity, shown in Table 7, is similar to the out of sample accuracy (95%).

**Table 6:** Consensus rules for the HCV NS3 data set.

HCV NS3 peptide set	
HCVA	P3 ∉ {K}
	P1 ∈ {C}
	P1' ∉ {P}

HCVB	P6 ∈ {A,N,D,C,E,I,K,M,F,P}
	P5 ∉ {I}
	P4 ∉ {N,D,Q,G,L,F,P,S,V}
	P3 ∈ {R,E,G,I,L,T,V}
	P1 ∉ {R,P,Y}
	P1' ∈ {A,R,N,D,H,M,S,W,Y}
	P3' ∉ {Q,E,I,L,M,P,V}

HCVC	P4 ∉ {S}
	P3 ∈ {R,E,G,I,F,T,V}
	P2 ∉ {C,L,S,T}
	P1 ∉ {R,D,P,Y}
	P1' ∉ {C,Q,E,G,I,L,K,P,T,V}
	P2' ∉ {P}
	P3' ∉ {P}
	P4' ∉ {A,R,D,E,G,I,P,S,T}

HCVD	P6 ∈ {C}
	P4 ∈ {R,C,E,I,M,T,V}
	P4' ∈ {C,W,Y,V}

Consensus rules for the HCV NS3 data set (939 peptides). The rules are listed in the priority order given by the OSRE method. The text in italics is the label for each rule.

**Table 7:** Fidelity for HCV NS3 rules.

Rules used	Accuracy
	HCV NS3

HCVA, HCVB, HCVC, HCVD	94.7%

The fidelity of the rules for the HCV NS3 data set.

#### Out-of-sample tests

Applying the HCV rules to the NS3 protease sequence (the NS3 internal test data set) produces 46 predicted internal cleavage sites, which are listed in Table 8. These can be compared to observed internal cleavages for the NS3 protease [32-34].

**Table 8:** The HCV NS3 rules applied to the NS3 protease sequence.

P1	Decamer	Rule match	Mass (kDa)	Mass (kDa)
C16	RGLLGCIITS	HCVA	1.7	65.6
C47	TFLATCINGV	HCVA	5.0	62.3
C52	CINGVCWTVY	HCVA, HCVC	5.5	61.8
Q73	KGPVIQMYTN	HCVC	7.7	59.6
M74	GPVIQMYTNV	HCVC	7.8	59.4
V78	QMYTNVDQDL	HCVC	8.3	59.0
C97	RSLTPCTCGS	HCVA	10.3	56.9
C99	LTPCTCGSSD	HCVA	10.5	56.7
S102	CTCGSSDLYL	HCVB	10.7	56.5
T108	DLYLVTRHAD	HCVB	11.4	55.8
G120	PVRRRGDSRG	HCVB	12.8	54.4
S122	RRRGDSRGSL	HCVC	13.0	54.2
I132	LSPRPISYLK	HCVC	14.1	53.1
G137	ISYLKGSSGG	HCVB	14.7	52.6
C159	FRAAVCTRGV	HCVA	16.7	50.5
V163	VCTRGVAKAV	HCVC	17.1	50.1
K165	TRGVAKAVDF	HCVC	17.3	49.9
Q195	PPAVPQSFQV	HCVC	20.6	46.6
A240	TLGFGAYMSK	HCVC	25.1	42.2
C279	LADGGCSGGA	HCVA	29.2	38.1
C289	YDIIICDECH	HCVA, HCVC	30.2	37.1
C292	IICDECHSTD	HCVA	30.5	36.7
G314	QAETAGARLV	HCVC	32.6	34.6
V339	PNIEEVALST	HCVB	35.2	32.0
L341	IEEVALSTTG	HCVB	35.4	31.9
C368	RHLIFCHSKK	HCVA, HCVC	38.3	28.9
N387	VALGINAVAY	HCVC	40.3	26.9
L395	AYYRGLDVSV	HCVB, HCVC	41.2	26.0
T402	VSVIPTSGDV	HCVC	41.9	25.3
C428	DSVIDCNTCV	HCVA, HCVC	44.5	22.7
C431	IDCNTCVTQT	HCVA	44.9	22.4
S488	SGMFDSSVLC	HCVC	51.2	16.1
C492	DSSVLCECYD	HCVA	51.6	15.7
C494	SVLCECYDAG	HCVA	51.8	15.4
A497	CECYDAGCAW	HCVD	52.1	15.1
C499	CYDAGCAWYE	HCVA	52.3	14.9
V511	PAETTVRLRA	HCVB	53.6	13.6
L513	ETTVRLRAYM	HCVB, HCVC	53.9	13.3
C525	PGLPVCQDHL	HCVA	55.2	12.0
V535	EFWEGVFTGL	HCVC	56.5	10.8
I542	TGLTHIDAHF	HCVC	57.2	10.0
Q552	LSQTKQSGEN	HCVC	58.4	8.8
A562	FPYLVAYQAT	HCVB	59.5	7.8
C568	YQATVCARAQ	HCVA, HCVC	60.1	7.1
C584	DQMWKCLIRL	HCVA	62.0	5.2
C622	KYIMTCMSAD	HCVA	66.3	1.0

The table shows the result of applying the HCV NS3 cleavage rules to the NS3 protease amino acid sequence. Only decamers that are predicted to be cleaved are listed. The first column mentions the amino acid and the position in the NS3 sequence that occupies the P1 position in the decamer. The second column lists the decamer. The third column shows which HCV NS3 rules that the decamer matches to. The fourth and fifth column show the molecular masses of the cleavage products if NS3 is cleaved in that position.

Yang et al. [32] report two internal cleavages of NS3 which are cleaved by the NS3 protease itself together with the NS4A co-factor. These are HLIFCH-SKKK (H369) and VSVIPT-SGDV (T402). The latter of these is predicted by the HCV rules (Table 8). The first one is not predicted to be cleaved but the nearby cleavage RHLIFC-HSKK (C368) is predicted. It is probably also the correct cleavage site (see discussion).

Kou et al. [33] tested the internal cleavages at HLIFCH-SKKK (which we believe is actually RHLIFC-HSKK) and VSVIPT-SGDV for sensitivity to genotype. They found that the latter cleavage had genotype specificity, i.e. NS3 protease from HCV-1b could not cleave NS3 protease from genotype HCV-2a, and vice versa. The HCV rules predict the same; NS3 from genotypes HCV-1a and HCV-1b is predicted to be cleaved at this site by the HCV rules, but not NS3 from genotypes HCV-2a, HCV-2b or HCV-2c.

Hou et al. [34] report an alternative cleavage of NS3/4A, which can be observed when the wild type NS3/4A cleavage site is mutated so that it is not cleaved. They estimate that the alternative cleavage site (an internal NS3 cleavage site) is located about 6 kDa upstream from the wild type cleavage site, based on their observations of two 12 kDa and 67 kDa fragments that appear when the wild type cleavage site is mutated, but they were unable to find the location of the alternative cleavage site although they tested to mutate sites that were 6 kDa upstream from the NS3/4A cleavage site [34]. It is therefore not possible to test whether the HCV rules would match this site. However, we can predict the possible cleavage sites under the assumption that the HCV rules are correct. It is peculiar that Hou et al. report observed masses for the NS3 and the NS3-NS4A complex which are larger (73 kDa and 80 kDa, respectively) than their molecular masses (which are 67 kDa and 73 kDa, respectively). It is possible that the reason is that there was something extra (with mass 6–7 kDa) sitting on the N-terminal side of the cloned NS3+NS4A complex, which was not cleaved off. This means that one should look for NS3 cleavage fragments of about 61 kDa and 6 kDa (since the NS4A has a mass just below 6 kDa). If this is correct then the alternative cleavage site could be in any of four positions (cf. Table 8): C47, C52, C568 or C584.

The HCV rules predict at least two correct cleavage sites (out of 46 potential sites), no false negatives and 575 (out of 621) true negatives for the NS3 internal test data. This indicates an out-of-sample specificity (true negative rate) of 93% and a sensitivity (true positive rate) of 100% (no false negatives) for the HCV rules.

The HCV rules produce 196 possible cleavage sites on the TLR3 test data set (2805 decamers). Of these, only one is a verified cleavage site, PSSTP-CSAHL in TICAM-1 [35], which matches a decamer in the HCV NS3 data set. None of the remaining 2804 decamers match any decamers in the HCV NS3 data set. Thus, there are (at most) 195 false positives and 2609 true negatives. This indicates an out-of-sample specificity of 2609/(195 + 2609) = 93%, which agrees with the estimate from the NS3 internal test data.

Kim et al. [36] performed an *in vivo *determination of HCV NS3 substrate specificity, using a genetic assay that produces random sequences based on the NS4B/5A cleavage site in HCV. They list 69 decamers that are especially good substrates for NS3 *in vivo*. Table 9 shows these 69 decamers (plus a consensus sequence suggested by Kim et al.) and how they match the HCV rules. Only one of the 69 decamers does not match any HCV rule. Most of the other decamers match the rule HCVA and many match more than one rule. This indicates an out-of-sample sensitivity of 68/(68 + 1) = 99%, which agrees well with the estimate from the NS3 internal test data.

**Table 9:** Applying the HCV NS3 rules to the NS3 in vivo test data.

Decamer	Rule match	Decamer	Rule match
DCYVYCSGSW	HCVA, HCVB, HCVC	DCSQPCAGSW	HCVA
DCAVTCSGSW	HCVA, HCVB	DCIIVCAGSW	HCVA, HCVB, HCVC
DCAVRCSGSW	HCVA, HCVB, HCVC	DCQQLCAGSW	HCVA
DCIKCCSGSW	-	DCPSPCAGSW	HCVA
DCVSNCSGSW	HCVA	DCAILCAGSW	HCVA, HCVB
DCVMKCSGSW	HCVA	DCIMPCAGSW	HCVA
DCATTCSGSW	HCVA, HCVB	DCVRMCAGSW	HCVA, HCVC
DCTQMCSGSW	HCVA	DCSVLCAGSW	HCVA
DCLDLCSGSW	HCVA	DCYRPCAGSW	HCVA, HCVB, HCVC
DCVTPCSGSW	HCVA, HCVC	DCLCLCAGSW	HCVA
DCVSLCSGSW	HCVA	DCAVCCAGSW	HCVA, HCVB
DCPLACSGSW	HCVA	DCPIMCAGSW	HCVA, HCVC
DCMCDCSGSW	HCVA	DCHQMCAGSW	HCVA
DCVLRCSGSW	HCVA	DCWTPCAGSW	HCVA, HCVB, HCVC
DCAVTCSGSW	HCVA, HCVB	DCIIKCCGSW	HCVA
DCSVRCSGSW	HCVA	DCIMECCGSW	HCVA
DCSHPCSGSW	HCVA	DCSQLCCGSW	HCVA
DCCVRCSGSW	HCVA, HCVB, HCVC	DCTVACTGSW	HCVA
DCSVLCSGSW	HCVA	DCLVACTGSW	HCVA
DCRVRCSGSW	HCVA, HCVB, HCVC	DCPCPCTGSW	HCVA
DCVTPCSGSW	HCVA, HCVC	DCLELCTGSW	HCVA
DCIYICSGSW	HCVA	DCLVACTGSW	HCVA
DCRLPCSGSW	HCVA, HCVB	DCQIICTGSW	HCVA
DCYERCSGSW	HCVA, HCVB, HCVC	DCVVCCGGSW	HCVA
DCAVLCSGSW	HCVA, HCVB	DCTVTTSGSW	HCVB
DCVRLCSGSW	HCVA	DCTVETSGSW	HCVB, HCVC
DCPTNCSGSW	HCVA, HCVC	DCSVVCSSSW	HCVA
DCPRLCSGSW	HCVA	DCSVACSQSW	HCVA
DCVSNCSGSW	HCVA	DCSTLCSTSW	HCVA
DCRIPCSGSW	HCVA, HCVB, HCVC	DCSITCAQSW	HCVA
DCVSNCSGSW	HCVA	DCSVLCARSW	HCVA
DCFAMCSGSW	HCVA	DCSVPCTGSW	HCVA
DCTIKCAGSW	HCVA, HCVB, HCVC	DCSLPCGSSW	HCVA
DCLITCAGSW	HCVA	DCSAPCGSSW	HCVA
DCKVTCAGSW	HCVA, HCVB	DCVVPCSGSW	HCVA, HCVC (*)

The table shows the result of applying the HCV NS3 cleavage rules to the 69 decamers listed as especially good substrates *in vivo *by Kim et al. [36]. The first and third column show the decamers and the second and fourth column show the HCV rules that it matches (if any). The last (70^*th*^) sequence (DCVVPCSGSW), marked with (*), is the consensus sequence tested by Kim et al., this sequence is already in the HCV NS3 data set and is thus not an out-of-sample prediction.

If the estimates of sensitivity and specificity are correct, then the positive likelihood ratio for the OSRE HCV rules would be 14.

## Discussion

### The artificial data

The results on the two artificial problems illustrate two things. First, OSRE quickly finds rules that are very close to the generating rules in terms of classification accuracy. Secondly, where an identified rule is not completely correct but the correction refers to rare events, then many examples are needed before the rare cases are represented in sufficient number to affect the performance ranking of the rule set and hence generate the required update.

The difficulty with artificial data set B lies in the very low probability for observing false positives once the rule (P2 ∈ {P} AND P1 ∈ {R}) has been learned. For example, the training data set with 100,000 observations has 50,002 positive examples and 49,998 negative examples. All the positive examples (of course) match the rule but only 97 of the negative examples (0.2% of the negative examples) match the rule and are thus counterexamples that point to that the rule must be modified.

In summary, OSRE finds a very good rule already with 100 samples and is able to find the corrections to this rule from a mere additional 97 false positives in the case of data set B. This must be considered a very good performance.

### HIV-1 PR data

Table 4 shows that the OSRE rules have a higher fidelity to the HIV-1 PR data sets than rules extracted using rough set theory [17], i.e. the OSRE rules are a more faithful description of the data used to generate them. The out-of-sample test, Figure 3, shows that the OSRE rules have excellent predictive power, matching that of the hitherto state-of-the-art predictor, the linear support vector machine [38], which is not a rule based method. This is a positive surprise since there is usually a trade-off between accuracy and comprehensibility. Other previously suggested predictors (rule based or not) have significantly worse accuracy than the OSRE rules and the linear support vector machine. These include a recently published HIV cleave web-server [37] and the rough set theory rules [17]. The rough set theory can produce better results if many more (hundreds of) rules are used but the comprehensibility is then definitely sacrificed [17]. The OSRE rules for HIV-1 protease are, on the other hand, both compact and accurate.

The rough set theory approach uses several physicochemical properties for the amino acids and one might believe that this should produce a better model than just using letter codes. The results here, however, indicate that this is not at all the case; conjunctive rules with simple amino acid letter codes are shorter, better predictors, and have higher fidelity to the data. Also, the OSRE method is fast; it will produce a small (comprehensible) rule set for the data in this study in the matter of minutes (the timing is discussed in the Methods section).

The OSRE rules for positions P1 and P1' are the most consistent rules and agree with earlier findings [15,39,40] for HIV-1 PR: large hydrophobic residues are preferred in position P1 and hydrophobic residues are preferred in position P1'. The OSRE consensus rules and the rough set rules do not agree completely. The rough set rules for cleaved peptides have, e.g., P3 ∈ {C} which does not occur very often in the OSRE consensus rules. Another difference is P1' ∈ {D,C,K} for the rough set rules but not for the OSRE rules. We cannot with certainty say whether the OSRE or the rough set rules are more correct, but the fact that the OSRE rules have a higher fidelity to the data sets indicates that the OSRE rules would be more correct.

The HIV-1 protease cleavage rules based on the 1625 data set, listed in in Table 3, require at least four residues to be specified for cleavage. This is different from Kontijevskis et al. [17], who find that three specified residues are sufficient. However, this is probably not a significant difference (the HIV-1 protease cleavage rules for the 746 data set contain one rule, HIVE1, where three positions are sufficient).

It is difficult, and hardly worthwhile, to compare the OSRE results for the HIV-1 PR data with other earlier rule extraction approaches, e.g. [18,20,41,42], except the rough set rule method. Earlier results have been based on a much smaller data set (362 peptides) and tend to refer to single amino acids in single positions and not groups of amino acids.

The two rules that agree most for the HIV-1 PR 746 and HIV-1 PR 1625 data sets are HIVA1 and HIVA2; 76% of the octamers that match HIVA1 also match HIVA2 and 69% of the octamers that match HIVA2 also match HIVA1, see Table 10. It is therefore reasonable to create a new consensus sequence by building a joint rule from HIVA1 and HIVA2, i.e. a rule that describes all octamers that match both HIVA1 and HIVA2. This consensus sequence is X-X- [ARNDCEGHIMFTWYV]- [LMFY]- [HILMFPYV]- [ACQEILMFTWYV]-X-X, where X denotes any amino acid. The sensitivity, specificity and positive likelihood ratio for this consensus sequence, when evaluated on the HIV-1 3261 PR data set, are 28%, 99% and 24, respectively. No other predictor is as accurate as this at this sensitivity level (cf. Table 5). It is notable that the consensus sequence only considers positions P2-P1-P1'-P2', i.e. not the full eight residue sequence.

**Table 10:** Mixing of HIV-1 rules.

HIV..	..A1	..B1	..C1	..D1	..E1	..A2	..B2	..C2	..D2	..E2
HIVA1	4.56%	1.90%	0.36%	0.27%	1.60%	3.45%	0.43%	0.41%	0.43%	0.29%
HIVB1		4.29%	0.31%	0.19%	0.93%	0.73%	0.53%	0.31%	0.16%	0.28%
HIVC1			3.23%	0.14%	0.12%	0.29%	0.24%	0.25%	0.25%	0.26%
HIVD1				2.05%	0.17%	0.28%	0.12%	0.31%	0.20%	0.22%
HIVE1					3.50%	1.58%	0.20%	0.17%	0.22%	0.12%

HIVA2						4.97%	0.54%	0.53%	0.67%	0.32%
HIVB2							2.56%	0.62%	0.22%	0.63%
HIVC2								1.86%	0.53%	0.55%
HIVD2									1.53%	0.41%
HIVE2										2.70%

There are 20^8 ^possible octamers.

The fractions of these octamers that match to each OSRE HIV-1 rule, and to more than one rule, are shown here. The diagonal elements in the table show the fraction of the possible octamers that match one rule. The off-diagonal elements in the table show what fraction of the octamers that match both rules.

### HCV NS3 data

A cysteine (C) in position P1 is considered to be the most important determinant for cleavage by HCV NS3/4A [31]. This is only reflected in the rule HCVA; the other rules are much less specific about the P1 position. Urbani et al. [27] conclude that the specificity is quite broad unless for the requirement for a small hydrophobic residue (e.g. C or T) in position P1. The OSRE HCV rule set indicates that the cysteine in position P1 is the dominating cleavage process: 10% of all decamers match the rule P1 ∈ {T,C}, 9% of all decamers match the rule HCVA and about 11% of all decamers match any of the OSRE HCV rules. This means that roughly 80% of the decamers that are cleaved by HCV NS3 (as predicted by the OSRE rules) match the rule HCVA.

The standard HCV NS3/4A cleavage rule [23,31,43] (P6 ∈ {E,D} AND P1 ∈ {T,C} AND P1' ∈ {A,S}) is much more specific than the OSRE HCV rules; only 0.1% of all possible decamers match this standard rule. Kim et al. [36] suggest an even more restrictive cleavage rule (consensus sequence) based on their *in vivo *studies: (P6 ∈ {E,D} AND P4 ∈ {V} AND P3 ∈ {L,P} AND P1 ∈ {C} AND P1' ∈ {A,S}). Only one in eight million decamers match this consensus sequence. None of the decamers studied by Kim et al. [36], except the one they handcraft to fit, match their consensus sequence.

None of the OSRE HCV rules support P6 ∈ {E,D} and P1' ∈ {A,S}. Thus, it cannot be said that the OSRE HCV rules are in strong agreement with previously reported consensus sequences. However, the OSRE HCV rules are more accurate at predicting the cleavage sites. They are dominated by HCVA and HCVC, which together match about 10% of all decamers, see Table 11. The two rules have quite a small overlap, 7% of the decamers that match HCVA also match HCVC, and 6% of the decamers that match HCVC also match HCVA (cf. Table 11). This indicates that there are two different processes taking place, one described by HCVA and the other described by HCVB and HCVC, which have a higher overlap. The contribution from HCVD is quite small and it can probably be ignored.

**Table 11:** Mixing of HCV NS3 rules.

	HCVA	HCVB	HCVC	HCVD
HCVA	4.51%	0.16%	0.33%	0.02%
HCVB		2.27%	0.77%	0.02%
HCVC			5.28%	0.04%
HCVD				0.35%

There are 20^10 ^possible decamers. The fractions of these decamers that match to each OSRE HCV NS3 rule, and to more than one rule, are shown here. The diagonal elements in the table show the fraction of the possible octamers that match one rule. The off-diagonal elements in the table show what fraction of the decamers that match both rules.

The positive likelihood ratio for the OSRE HCV rules is estimated to be 0.99/(1-0.93) *≈ *14, which is the same as for the HIV-1 PR consensus sequence, but the sensitivity is much higher for HCV NS3/4A than for HIV-1 PR.

It was mentioned in the result section that we believe the internal cleavage of NS3 to be at C368 (RHLIFC-HSKK) and not at H369 (HLIFCH-SKKK) that Yang et al. [32] report. There are two reasons for this. One is that the HCV rules match one site and not the other. The other reason is that the experimental data presented by Yang et al. fits also with a cleavage at C368. They [32] test their predicted location of the cleavage site by several pairwise mutations in the HCV polyprotein: ...RHLIFCHSKKKC... → ...RHLPGCHSKKKC... (pNS34A-M4); ...RHLIFCPGKKKC... (pNS34A-M5); ...RHLIFCHSKPGC... (pNS34A-M6); and ...RHLIFCHSKKPG... (pNS34A-M7). Yang et al. note that the internal cleavage is blocked when the mutation is pNS34A-M5, whereas the internal cleavage occurs in all other cases. These observations are perfectly consistent with cleavage at C368 (between C and H) if the rule HCVA is correct.

It is not worthwhile to compare the OSRE HCV rules to previous automated rule extraction (and prediction) work on HCV NS3 protease since these have been based on a data set with many errors in it.

The HCV NS3 problem seems to be easier than the HIV-1 PR problem. Fewer rules are required and the OSRE NS3 rules are more accurate than the OSRE HIV-1 PR rules.

## Conclusion

A methodology that combines the OSRE rule extraction method and spectral clustering was introduced for efficiently, i.e. quickly and accurately, extracting accurate and comprehensible specificity rules for proteases, rules that also had a high fidelity to the data used to create them. The approach was demonstrated on two medically important protease cases, HIV-1 protease and HCV NS3/4A protease. The HIV-1 protease rules were shown to be more accurate than previous state-of-the-art rule extraction results on a large HIV-1 protease out-of-sample test data set. The proposed methodology achieved this performance using fewer rules than previous approaches and with a higher fidelity to the data set that had been used to create the rules.

The HCV NS3/4A protease rules were shown to fit very well with experimental findings for the HCV NS3 protease. The rules were used to correct the position of an internal cleavage site in HCV NS3, which demonstrates the usefulness of accurate and comprehensible rules when interpreting experimental data.

The results indicate that the HCV NS3/4A protease cleavage is a simpler problem than the HIV-1 protease cleavage.

In summary, the OSRE approach yields rules that are simpler and more accurate than other rule extraction methods for protease specificity problems, and it is significantly faster. It does so by using conjunctive low-order rules, i.e. rules with few arguments and in a form that is commonly used to describe protease specificities.

## Methods

### Orthogonal search-based rule extraction (OSRE)

The OSRE algorithm [44] finds conjunctive rules for classifications from any classifier that produces a smooth response surface. It is an efficient method to find low-order rules from labeled data. Given a data set with an associated classification label, the method starts by fitting the response surface that best classifies the data. This is a smooth surface in data space which separates it into different regions by means of generic, non-linear decision boundaries. This fit to the data must be obtained by a robust statistical methodology, to ensure good out-of-sample generalization [44]. This step is essential in order to obtain a smooth fit of the data, cutting through noise and thus avoiding over-fitting. In the first stage of the rule-extraction algorithm, rules are fitted to the response surface of this statistical model, rather than to the data themselves. For this reason, the non-linear model is a multi-layer perceptron neural network with strong regularization using the Bayesian framework of Automatic Relevance Determination [45]. In cases where the data are linearly separable, this heavy robust regularization will default to a linear decision boundary. Cycling through the data, this stage results in the maximal multivariate box, centered on each data point, which is to one side of the decision boundary, returning an initial number of rules equal to the number of data points.

The second stage in the application of OSRE, is to sort the rules by their performance on the data set, measuring performance by the proportion of actual data points within each multivariate box, i.e. conjunctive rule, which belongs in the correct class. This process includes removing boxes within boxes and selecting the rules with the best balance between coverage, i.e. true detection rate, and specificity, i.e. a low false detection rate. This is achieved by starting with the individual rule whose performance in the ROC plot is closest to the ideal point with unit sensitivity and specificity, then adding more rules in a stepwise manner, each time selecting the best additional rule by measuring the position of the aggregate rule set in the ROC plot. The automatic forward selection of the minimal rule set that best approximates the ideal performance of unit sensitivity and specificity is a development of the OSRE methodology since the publication of the original paper.

By not imposing a requirement of mutual exclusivity between individual rules and, instead, searching directly in a multivariate space, rather than in a sequential univariate manner as with most rule extraction methods, it is found that the well performing rules are of low-order. This means that the rules are more readily interpretable by expert users, as they involve fewer arguments. The representation framework using multivariate conjunctive rules at each node in the search hierarchy, rather than univariate nodes in a decision tree, is a particular feature of the OSRE methodology which makes it more suitable to the derivation of rule sets that are interpretable by human experts. In effect, it represents a trade-off between the simplicity in the definition of each node in the tree, which is now a multivariate vector rather than a scalar node, for simplicity of the rule set as whole, with fewer and simpler rules.

The method was originally validated on artificial data [44] and has since been successfully applied in a number of practical applications, e.g. [46].

Creating the decision surface is what tends to take the longest time. This is, however, sometimes available from a previous study. The rule extraction phases (first and second stages) scale, in the current implementation, approximately quadratically with the number of observations. Running OSRE on HIV-1 data sets of different sizes, excluding the decision surface creation, takes approximately 0.5 seconds for 100 observations, 12 seconds for 1600 observations, and 60 seconds for 3200 observations (on an AMD Athlon 64 Processor 2 GHz 3 GB RAM with Windows XP SP3). These are indicative times and individual runs may vary a lot.

### Model validation

The OSRE method was applied using ten-fold cross-validation (CV) on the data sets. This means that the OSRE method extracted rules using 90% of the data and the rules were then tested on the remaining 10%, which thus was a hold-out sample. This was repeated ten times so that each peptide in the data set had been used out-of-sample once. The average out-of-sample accuracy over these ten runs was then used as the expected generalization performance of the OSRE method.

The performance obtained by OSRE on the cleavage data was obtained by the application of the standard methodology as outlined above, rather than by selecting the best performance from a range of possible methodologies, which may be biased towards the suitability of the chosen method to the particular nuances of the data sets under study. Therefore, it is expected that this performance benefit represents a generic feature of the proposed methodology, which will generalize to other cleavage data.

### Rule clustering

OSRE produced slightly different rules for each CV subset. The rules were therefore grouped using the normalized cut spectral clustering algorithm [47]. The affinity matrix **W **used had elements



where *d*_*ij *_was the fraction of peptides that the two rules disagreed on when evaluated on the data set that the rules were extracted for (i.e. the 1625 HIV-1 PR data set, the 746 HIV-1 PR data set or the 939 HCV NS3 data set). The number of clusters was set to five, which was a subjective choice based on the OSRE results and the quality of the clusterings measured with the Fowlkes-Mallows index [48]. The iterative clustering method gave slightly different results each time it was run. It was therefore repeated five times and the rules that clustered together all five times were grouped together. The most common amino acids in each position within each group then defined the consensus rule for that group. Two of the five clusters mixed very much for the HCV NS3 data. These two clusters where joined and there are therefore only four consensus rules for the HCV NS3 data although the clustering was done with five clusters.

The fidelity of the consensus rules was tested on the data after the clustering had been done. The consensus rules (and the number of clusters) were not changed once the rules had been tested on the data.

## List of abbreviations

A Ala: Alanine; AIDS: Acquired Immune Deficiency Syndrome; CTL: Cytotoxic T Lymphocyte; C Cys: Cysteine; CV: Cross Validation; D Asp: Aspartate; E Glu: Glutamate; F Phe: Phenylalanine; G Gly: Glycine; H His: Histidine; HCV: Hepatitis C Virus; HIV-1: Human Immunodeficiency Virus type 1; IKK*ε*: I*κ*B kinase *ε*; I Ile: Isoleucine; K Lys: Lysine; L Leu: Leucine; M Met: Methionine; N Asn: Asparagine; NS3: Non Structural Protein 3; NS4A: Non Structural Protein 4A; OSRE: Orthogonal Search-based Rule Extraction; P Pro: Proline; Q Gln: Glutamine; R Arg: Arginine; ROC: receiver operator characteristic; S Ser: Serine; T Thr: Threonine; TBK1: TRAF family member-associated NF-*κ*B activator-binding kinase 1; TLR: Toll-like receptor 3; TRIF TICAM1: Toll-IL-1 receptor domain-containing adaptor inducing IFN-*β*; V Val: Valine; W Trp: Tryptophan; Y Tyr: Tyrosine.

## Authors' contributions

TR designed the study, did the rule clustering work, some analysis and drafted the manuscript. TAE implemented the OSRE methodology. LY collected the data, produced some of the results (rough set rules test and state-of-the-art classifiers) and did some of the analysis. DG contributed to the collection of data and interpretation of the results. TAE, IJH and PJGL converted the data into a form compatible with the OSRE methodology and extracted the OSRE rules from the 10 fold cross validation data sets. All authors contributed to the writing and revised the manuscript for intellectual content and approved the final version of the manuscript.
